# A Novel Hybrid High-Speed Mass Spectrometer Allows Rapid Translation From Biomarker Candidates to Targeted Clinical Tests Using ^15^N-Labeled Proteins

**DOI:** 10.1016/j.mcpro.2025.101050

**Published:** 2025-08-13

**Authors:** Maria Wahle, Philip M. Remes, Vincent Albrecht, Michael Baggio Lorenz, Johannes Mueller-Reif, Sophia Steigerwald, Tim Heymann, Lili Niu, Philip Lössl, Stevan Horning, Cristina C. Jacob, Matthias Mann

**Affiliations:** 1 Department for Proteomics and Signal Transduction, Max-Planck Institute of Biochemistry, Martinsried, Germany; 2 Thermo Fisher Scientific, San Jose, California, USA; 3 Proteomics Program, Novo Nordisk Foundation Center for Protein Research, Faculty of Health and Medical Sciences, University of Copenhagen, Copenhagen, Denmark; 4 Absea Biotechnology GmbH, Berlin, Germany; 5 Thermo Fisher Scientific (Bremen), GmbH, Bremen, Germany

## Abstract

Recent developments in affinity binder or mass spectrometry (MS)-based plasma proteomics are now producing panels of potential biomarker candidates for diagnosis or prognosis. However, clinical validation and implementation of these biomarkers remain limited by the reliance on dated triple quadrupole MS technology. Here, we evaluate a novel hybrid high-speed mass spectrometer, Stellar MS, which integrates the robustness of triple quadrupoles with the enhanced capabilities of an advanced linear ion trap analyzer. This instrument allows for extremely rapid and sensitive parallel reaction monitoring (PRM) and MS3 targeting. The Stellar MS allowed targeting thousands of peptides originally measured on Orbitrap Astral MS, achieving high reproducibility and low coefficients of variation (CV) as well as sensitivity and specificity sufficient for many of the top 1000 plasma proteins. Furthermore, we developed targeted assays for alcohol-related liver disease (ALD) biomarkers, showcasing the potential of Stellar MS in clinical applications. Absolute quantification is typically a requirement for clinical assays, and we explore the use of ^15^N-labeled protein standards in a rapid, streamlined, and generic manner. Our results indicate that the Stellar MS can bridge the gap between proteomics discovery and routine clinical testing, enhancing the diagnostic and prognostic utility of protein biomarkers.

## Highlights

• Introducing the Stellar MS for biomarkers in plasma proteomics.
• Translation of discovery DIA data into targeted PRM assays.
• Targeting thousands of peptides in short gradients.
• Evaluation of 15N labeled protein standards in neat plasma.

## Graphical Abstract

**Figure:**
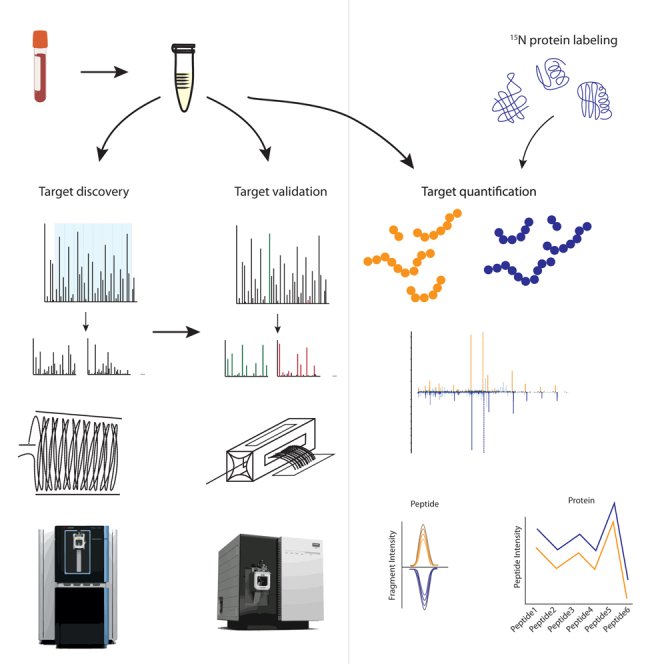

## In Brief

The novel Stellar mass spectrometer bridges the gap between biomarker discovery and clinical implementation. By combining the robustness of triple quadrupole instruments with the enhanced capabilities of an advanced linear ion trap, it shows efficient transfer of discovery data to targeted assays. Their innovative use of ^15^N-labeled protein standards enables targeting multiple peptides per protein simultaneously, improving reliability and sensitivity. This practical approach provides a foundation for translating proteomics discoveries into clinical applications, with potential impact beyond plasma proteoamics.

The field of mass spectrometry (MS)-based plasma proteomics has experienced remarkable advancements in recent years (1, 2, 3). Technological innovations and improvements in sample preparation workflows have expanded our ability to profile the plasma proteome in greater depth. This now enables discovery-based studies that consistently demonstrate the diagnostic potential of proteomics, which is starting to surpass the capabilities of current clinical assays.

Despite these advances, clinical laboratories predominantly continue to rely on antibody-based methods, such as ELISA, which focus on single protein targets. In contrast, targeted MS can simultaneously evaluate multiple protein targets and is routinely used in newborn screening, therapeutic drug monitoring, and hormone assays, demonstrating that MS-based methods can achieve regulatory and clinical acceptance.

Triple quadrupole mass spectrometers, known for their simple yet effective architecture, remain the gold standard for targeted protein biomarker measurements. These instruments provide high signal-to-noise ratios and exceptional robustness, essential for reliable clinical diagnostics and are readily used with isotope labeled standards for absolute quantification (4, 5). However, they are restricted to selected or multiple reaction monitoring (SRM/MRM), instead of comprehensive MS/MS spectra (6). This limitation hampers the translation of proteomics discoveries into clinical applications, which typically takes many months even if sufficient specificity and sensitivity are achieved. Rare exceptions prove the point, as in the successful transfer of a targeted thyroglobulin quantification assay, which lacks reliable ELISA alternatives (7, 8).

Liver disease is a growing public health concern given that more than one-third of the world's population has steatotic liver disease, a substantial proportion of which will progress to steatohepatitis, fibrosis, and more severe, even life-threatening conditions (9, 10, 11). As the disease is largely asymptomatic, cost-effective, and specific tests are urgently needed. In this context, we recently described protein biomarker panels with high prognostic and diagnostic potential for the diagnosis of different stages of alcohol-related liver disease (ALD), which matched or outperformed all of the test procedures in routine clinical use (12). However, these promising results have yet to be translated into standardized clinical tests.

Here, we explore a novel hybrid high-speed mass spectrometer (Stellar MS) to begin to address the above challenges. The Thermo Scientific Stellar MS is designed to combine the simplicity of triple quadrupoles with the advantages of an advanced dual-pressure linear ion trap as the final mass analyzer. In contrast to the last quadrupole of a triple quadruple, the linear ion trap captures all fragments of the peptide ions of interest, and then rapidly scans out their entire spectrum without loss. This results in a platform with familiar technologies, performing an experiment that is already well-accepted in the clinic, but at a much increased scale and with the quantitative and qualitative benefits inherent to parallel fragment accumulation and full scan mass analysis. In the context of targeted mass spectrometry, this promises unprecedented sensitivity, specificity, and speed, including parallel reaction monitoring (PRM) as well as MS3 capabilities.

Furthermore, we introduce the use of full-length ^15^N-labeled protein standards in this context. Unlike peptide-based isotopic labeling, these standards control for variability in digestion and sample preparation, offering improved quantification accuracy. The ^15^N labeling also reduces potential interferences in MS2 spectra, increasing the reliability of fragment ion quantification (13). This approach holds promise for detecting proteoforms and post-translational modifications, enhancing the diagnostic potential of protein biomarkers.

In this article, we first compare the Stellar MS to established triple quadrupole technology and then describe how to transfer peptide panels from the Thermo Scientific Orbitrap Astral instrument to targeted assays, making use of the Stellar MS's adaptive real time retention time realignment. We find that essentially all Orbitrap Astral identified plasma peptides can successfully be targeted and describe how to prioritize the most promising ones. Finally, we employ a panel of ^15^N-labeled protein biomarkers for liver disease to advance the prospect of a universal clinical liver disease test.

## Experimental Procedures

### Plasma Collection

The patient plasma was collected by the Ludwigs-Maximilian-Universität München in accordance with the 1975 Declaration of Helsinki as approved by the LMU Ethics committee. Briefly, plasma is collected from donors using EDTA tubes (BD Vacutainer K2E; REF 367525). After collection the tubes were inverted three times and centrifuged at 2000*g* for 20 min at 4 °C. The plasma was separated, pooled, aliquoted, snap-frozen and stored for several years at −80 °C.

### Plasma Sample Preparation

Plasma samples were prepared based on the previously published plasma proteome-profiling pipeline (14). Shortly, 45 μl of 100 mM Tris (pH 8.0) was added to 5 μl of plasma for a tenfold dilution and mixed thoroughly. Ten microliters of diluted plasma were transferred into 10 μl of reduction/alkylation buffer (20 mM tris(2-carboxyethyl)phosphine (TCEP), 80 mM chloroacetic acid (CAA)). The digestion mix was shortly centrifuged up to 500*g* and heated to 99 °C for 10 min, followed by cooling to room temperature for 2 min in a PCR cycler. For digestion of the denatured proteins, 20 μl of a freshly prepared digestion mix (0.025 μg/μl trypsin and LysC in ddH2O) was added for a final digestion volume of 40 μl, followed by incubation at 37 °C, shaking (1000 rpm) overnight. Following overnight digestion, the enzymatic activity was quenched by adding 60 μl of 0.2% trifluoroacetic acid (TFA). For mass spectrometry (MS) measurement, 500 ng (1 μl) or 250 ng (5 uL of a 1:10 dilution) of the digested peptides were loaded onto disposable Evotip C18 trap columns (Evosep Biosystems) according to the manufacturer's instructions. Shortly, Evotips were soaked in 1-propanol and then activated with 0.1% formic acid (FA) in acetonitrile (ACN), centrifuging at 700*g* for 1 min. This was followed by renewed soaking in 1-propanol and a washing step using 0.1% FA in water. To ensure proper sample loading, 100 μl of 0.2% TFA was added to the Evotips and 1 μl of sample was added into the solution before centrifugation at 700*g* for 1 min. Evotips were then washed with 0.1% FA before adding 150 μl 0.1% FA to prevent drying of the C18 material. Evotips were kept at 4 °C until measurement and digested samples were stored at −20 °C.

### ^15^N-Labeled Protein Preparation

^15^N-labeled proteins listed in Supplemental Table S1 were obtained from Absea Biotechnology GmbH (Berlin, Germany). The constructs were cloned into pET30a vectors and expressed as His-tagged proteins in E.coli BL21(DE3) cultured in ^15^N-supplemented M9 medium. The proteins were purified by immobilized-metal affinity chromatography and stored in phosphate-buffered saline (pH 7.4). Protein purity and absolute concentration were assessed by SDS-PAGE and amino acid analysis (AAA). The proteins were either spiked into plasma and digested together (as described above) or digested separately before spike-in. For separate digestion, the proteins were diluted in a TEAB-based buffer (60 mM TEAB, 10% ACN, 10 mM TCEP, 40 mM CAA). After denaturation, reduction, and alkylation for 10 min at 74 °C, followed by cooling to room temperature, trypsin and LysC were added at a 1:25 ratio, and the proteins were digested overnight at 37 °C. The digestion was stopped by acidification to 1% TFA, and the digest was either stored at −20 °C or directly loaded onto Evotips as described above. Digests were either loaded in triplicate with 1 ng per digested protein or loaded as quadruplicates in a 1:4 dilution series starting from 25 ng. Here, a stock digest at a concentration of 25 ng/μl ^15^N protein (per construct) in human plasma was diluted using a ^15^N-free digest from the same plasma pool.

### DDA and DIA LC-MS Acquisition on Orbitrap Astral MS

Plasma samples as well as the ^15^N labeled proteins were analyzed using the Evosep One liquid chromatography (LC) system (Evosep Biosystems) coupled to an Orbitrap Astral mass spectrometer (Thermo Fisher Scientific). Peptides were eluted from the Evotips with up to 35% ACN and separated on an 8 cm ionOptics Aurora Rapid column (8 cm, 150 μm ID, 1.7 μm C18) at 50 °C using the Evosep 60 samples per day (SPD) method (21 min) or on a 5 cm ionOpticks Aurora Rapid column (5 cm, 75 μm ID, 1.7 μm C18) at 60 °C using the Evosep whisper zoom 80 SPD method. The mass spectrometer is interfaced with an EASY-Spray Source equipped with a FAIMS Pro device (Thermo Fisher Scientific) with the emitter held at 1900 V and a total carrier gas flow of 3.5 L/min at a FAIMS CV of −40 V.

Data-dependent acquisition (DDA) data were only used to assess the labeling efficiency of the ^15^N protein constructs; here, a mix of 1 ng protein per construct was separated using the Evosep Whisper 80 gradient. Full MS scans from 380 to 1650 m/z were acquired at a resolution of 240,000 at m/z of 200 with a normalized automatic gain control (AGC) target of 300% and a maximum injection time of 25 ms. MS/MS scans were acquired with the Astral detector using a topN = 50 method with a normalized AGC target of 100% and a maximum injection time of 10 ms. Only precursors with charge states between 2+ to 6+ were selected for sequencing, and the precursor isolation windows were set to 1.4 Thomson (Th). Previously, target precursors were excluded from (re-)sequencing for 6 s. The normalized collision energy for higher-energy collisional dissociation (HCD) fragmentation was set to 25%.

Plasma samples as well as ^15^N spike-in samples were acquired in data-independent acquisition (DIA) mode. The MS1 spectra were recorded using the Orbitrap analyzer at 240k resolution (200 m/z) from m/z 380 to 980 using an automatic gain control (AGC) target of 500% and a maximum injection time of 3 ms. The Astral analyzer was used for MS/MS scans in data-independent mode with three Th non-overlapping isolation windows with a scan range of 380 to 980 m/z. The precursor accumulation time was 5 ms, and the AGC target was 500%. The isolated ions were fragmented using HCD with 25% normalized collision energy.

### DIA and Targeted LC-MS Acquisition on Stellar MS

We used the Stellar MS with the same upfront setup as the Orbitrap Astral MS described above. In brief, peptides are separated over an 8 cm IonOpticks Aurora rapid column using the Evosep One system with a 60SPD gradient. The mass spectrometer is interfaced with an Easy-Spray source coupled with a FAIMS Pro device at a FAIMS CV of −40V.

DIA experiments were acquired with the gas phase fractionation (GPF) approach. Here, six replicates cover the range from 380 to 980 m/z in steps of 100 m/z per replicate. MS1 spectra are acquired for precursor mass ranges m/z 380 to 480, 480 to 580, …, 880 to 980 at a scan rate of 67 kDa/s and at an absolute AGC target of 30.000. MS/MS scans are recorded at the matching mass ranges using a 1 Th isolation window with a maximum injection time of 15 ms and an absolute AGC target of 1e4 charges (100%). Fragments are generated at an HCD of 30% and analyzed over a scan range of 200—1000 m/z at a scan rate of 125 kDa/s. All methods included an adaptive RT DIA experiment, which was set to acquire a reference.

Targeted MS2 assays were created from the same base method setup. Unless further specified, the targeting methods only differ in their target list. All targeted assays included an adaptive RT experiment, where, for the first set of experiments, an Orbitrap Astral-based rtbin file was used as a reference (definition is described below), exchanged with one generated during the first set of experiments for all following acquisitions of the same sample type. MS1 spectra were recorded over a range of 350 to 1250 m/z at a scan rate of 125 kDa/s with an AGC target of 30.000. Targeted MS/MS scans are acquired using a 1Th isolation window with a normalized HCD of 27% at an AGC target of 10.000 with the maximum injection time mode set to “Dynamic”. Precursors are targeted between the start and end time points defined in the target list. The aspired cycle time setting differs depending on the number of targets per run aimed toward yielding seven or more datapoints per peak. Targeted MS3 scans are acquired using the same settings as described for targeted MS/MS assays, including a second fragmentation step with a CID of 35% at 2 ms activation time and q = 0.22. Note that currently, CID for the second activation stage is done serially for each product in a high-to-low-m/z fashion, which has very high fragmentation efficiency and capture but costs an additional 2 ms per product ion. The second isolation stage in the ion trap uses a calibrated isolation width that depends on the relative mass-to-charge values of the multiplexed MS3 precursors, and ranges from a minimum of 2 Th for the lowest m/z precursor in a set, up to a maximum of around 10 Th for precursors that are more than 4x the m/z of the lowest precursor ion.

### Targeted Precursor Selection Using Skyline and PRM Conductor

Targeted assays were created based on Orbitrap Astral or Stellar MS peptide search results. A detailed description of the search parameters is described below.

The initial targeting list for the ^15^N-labeling-based assay was generated based on the search results of an unlabeled DIA-NN search, where the labeled peptide counterparts were added through Skyline. With the PRM Conductor, all precursors were targeted with an optimized acquisition window and a maximum time window of 2.5 min. The results were used to refine the targeting assay by removing targets not encoded in the 15N construct or not targetable in both channels, followed by reducing the acquisition window to 2 min per precursor while increasing the datapoints per peak to 12. Only precursors with a minimum absolute area of 100, a signal-to-noise ratio of two, and a minimum time correlation of 0.75 were retained. The same settings were used as a base for creating the MS3 assay. When activating MS3 as the acquisition type, PRM Conductor uses a heuristic for selecting multiple MS2 fragments to become multiplex-isolated MS3 precursor ions. Briefly, the highest intensity product ions are selected to satisfy the criteria that they are either multiply charged or if singly charged, they have a mass greater than the intact precursor.

Label-free targeted assays were created based on either Orbitrap Astral DIA or Stellar MS DIA-GPF peptide search results. Search results for both Orbitrap Astral and Stellar MS were imported into Skyline, and PRM Conductor was used to create a set of targeted assays, where the “Keep all precs.” Option was selected. This resulted in six initial assays for the Orbitrap Astral-based library and three assays for the Stellar MS-based library with 2 min scheduled acquisition windows. For both Orbitrap Astral and Stellar MS candidate targets, three replicates were acquired. The targets having CV greater than 30% were removed, and then PRM Conductor was used to select the precursors having at least three transitions satisfying the criteria of minimum absolute area 100, minimum signal-to-background ratio 2.0, minimum relative area 0.05, minimum time correlation to the median peptide chromatogram 0.80, and LC peak width between 4.0 and 20.0 s. This reduced the target list down to three intermediate assays for the Orbitrap Astral-based setup while generating the final target list for the Stellar MS-based assay. The three intermediate assays were filtered with the same settings, this time activating PRM Conductors' “Balance Load” function, leading to only one assay being generated.

Adaptive RT realignment was utilized in the targeted assays based on reference data from the discovery runs. To create reference spectra for aligning to Orbitrap Astral discovery, the 3 Th isolation width spectra were combined in silico to create spectra having an effective 51 Th isolation width, using the “Combine DIA Windows for Reference” option in PRM Conductor. The Stellar MS GPF experiments already contained an extra set of 50 Th isolation width acquisitions to gather reference data.

### Raw Data Analysis

The Orbitrap Astral raw files acquired in DDA mode were analyzed using Fragpipe 22.0 with the default workflow. In brief, peptides were generated from a reviewed Swiss-Prot human FASTA including common contaminants (from 01/31/2023), resulting in 20,437 protein entries, digested with a strict tryptic rule (cuts always after K/R), allowing for a maximum of two missed cleavages. Methionine oxidation, carbamidomethylation, and N-terminal acetylation were set as variable modifications with a maximum of one each per sequence, and ^15^N was included as a fixed modification for each amino acid. The initial precursor and fragment mass tolerances were set to 20 ppm, which were optimized automatically during the search. The search results were filtered with a protein and precursor FDR of 1%.

The precursor-based labeling efficiency of each protein was estimated by extracting the MS1 raw intensities wherever a precursor was matched using alphaRaw (15). The extracted raw intensity ratio of the (M-1/M0) was compared to predicted raw intensity ratios for different ^15^N contents (from 100% to 95%) for each precursor generated using alphaBase (15).

DIA raw files from Orbitrap Astral were analyzed using DIA-NN 1.9.1 with a spectral library generated by DIA-NN in library-free mode from a reviewed Swiss-Prot human FASTA including isoforms (06/17/2024) containing 82,112 proteins digested with Trypsin/P and a maximum of one missed cleavage with N-terminal methionine excision and carbamidomethylation as a fixed modification activated. MS1 and MS2 tolerances are inferred automatically, and results are filtered with a protein and precursor FDR of 1%.

The Stellar MS DIA search was performed with the CHIMERYS intelligent search algorithm (MSAID GmbH, Germany) in Thermo Scientific Proteome Discoverer Software 3.1 with fragment tolerance of 0.5 Da, using a reviewed Swiss-Prot human FASTA (04/25/2019) with 20,416 entries digested with a tryptic rule and a maximum of two missed cleavages allowed with carbamidomethylation set as a fixed modification and Methionine oxidation set as a variable modification (maximum of three allowed). Results were filtered to an FDR of 1%.

Raw files going directly into the targeted method generation were analyzed as described above. Targeted raw files were analyzed using Skyline-daily version 24.1.1.284.

For the label-free targeted assays, Skyline-daily's settings, like peak picking or peak boundaries, were not modified. Peak Area coefficient of variations (CVs) are reported using the default normalization strategy. Results are exported with Skyline's report option and visualized using Python.

The ^15^N-labeled targeted assays were manually curated for each precursor. Peak boundaries and fragments for the LOD/LOQ estimation were evaluated and adjusted based on the retention time and peak integration boundaries for the ^15^N-labeled precursor in the highest concentration. Results are exported, and LOQs are calculated with a bilinear regression fit with a one/x regression weighting and total ion current normalization and a maximum LOQ CV of 20%. The LODs were calculated using the bilinear turning point (16). This method is implemented as an experimental feature in the Skyline daily version; therefore, for absolute quantification, each precursor quality was assessed manually again.

Absolute quantification was performed based on the same set of fragments used in the LOQ estimation. Peak boundaries were adjusted manually, and for each precursor, the individual fragment areas were exported and analyzed using Python. Precursors with a per-fragment and label-based CV above 20% were excluded for quantification. Subsequently, each precursor intensity was calculated as the sum of the fragment intensities, and the replicates were summarized as the mean intensity per precursor. The precursor-based mean heavy intensity was corrected based on the estimated labeling efficiency of matching precursors. These mean intensities were used to calculate the light/heavy ratios and subsequently the absolute amount of light precursor. If the calculated amount of light precursor was below the LOQ, the precursor was excluded from absolute quantification. In addition, each precursor pair's extracted ion chromatogram was evaluated to exclude a heavy signal below a minimum signal-to-noise ratio of 5, or precursors that had less than three interference-free fragments. The absolute quantity of each protein was calculated based on the mean absolute quantity of each quantified precursor pair.

### Experimental Design and Statistical Rationale

All experiments were done using human plasma obtained as described above. Altogether, the dataset, including raw data files and search results, was uploaded to MassIVE and Panorama (see below). We used the same plasma batch for benchmarking and technical evaluation. In brief, measurements with different gradients and input amounts were done in triplicate unless mentioned otherwise. The experimental design and statistical rationale are described in the respective figure legends. Reproducibility and quantitative accuracy were evaluated using technical replicates. Proteins targeted in the ^15^N-labeled assay were selected from a proposed biomarker panel described in a previous publication (12) based on their production and purification efficiency.

### Development and Analytical Validation of Targeted MS Measurements

All experiments fall within the category of a Tier three targeted study. The data was acquired using PRM and transitions were selected based on experimental data. Quantitative information is retrieved using the summed peak areas as selected by Skyline.

## Results

### Principle of the Novel Hybrid Mass Spectrometer in Comparison to a Triple Quadrupole MS

The Stellar MS is a unique derivative of the Thermo Scientific Orbitrap Fusion Tribrid and TSQ Altis Plus mass spectrometer. It utilizes the Orbitrap Fusion Tribrid instrument control software, and the chassis, vacuum chamber, turbo pump, and quadrupole mass filter of the TSQ Altis Plus MS. The ion funnel, Q00 with low-pass-filtering capabilities, and dual-cell linear ion trap are identical to those used in the Thermo Scientific Orbitrap Ascend Tribrid MS, while the Q0 and Q2 multipoles were redesigned for Stellar MS. The linear ion trap RF system on the Stellar MS achieves a slightly higher frequency of 1260 kHz, up from around 1180 kHz. The dual-high-energy-dynode-single-multiplier, capable of single ion detection, used in the Orbitrap Ascend Tribrid MS, is combined with a new photo-multiplier-based final detector stage, as used in the Orbitrap Astral MS, that considerably increases the detector stability and longevity.

**Fig. 1:**
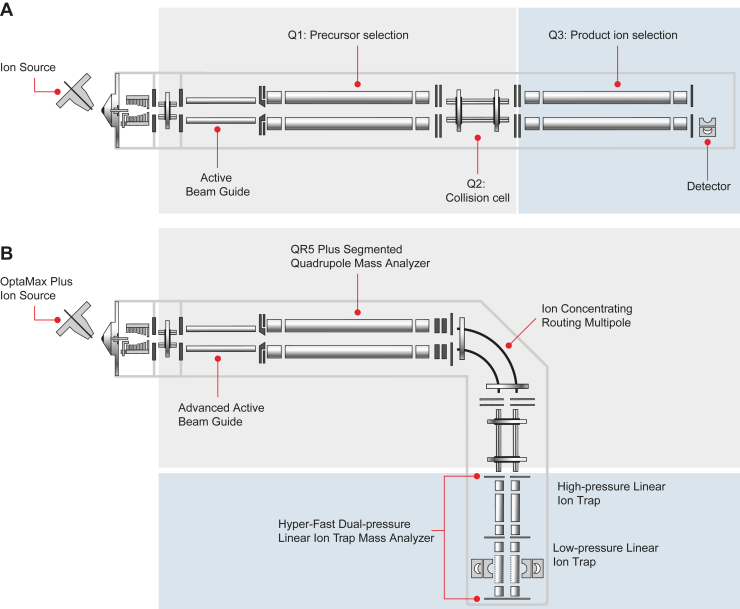
**Comparison of traditional triple quadrupole mass spectrometer (MS) and Stellar MS**. *A*, schematic of a traditional triple quadrupole MS, showing Q1 for precursor ion selection, Q2 for ion fragmentation, and Q3 for product ion selection. This setup limits the number of transitions to one (SRM) or a few, typically 3 to 5 (MRM). *B*, schematic of the Stellar MS, featuring a dual-cell linear ion trap (LIT) instead of Q3. The LIT allows simultaneous ion accumulation and fragmentation, achieving acquisition rates of about 70 Hz and enabling MS3 analysis at up to 40 Hz. This enhances sensitivity and specificity compared to traditional SRM. Note that the only conceptual difference consists of replacing Q3 with the LIT (shaded *blue* in the figure), thus robustness and usability should be similar.

Clinical targeted assays are typically designed for and run on triple quadrupole mass spectrometers. Conceptually, their layout is simple, starting with the precursor selection in the first quadrupole, followed by fragmentation in the second quadrupole and product ion (transition) selection in the third quadrupole (Fig. 1*A*). Given that only one fragment ion can be measured at a time, the number of transitions that can be measured at any given time is limited. Acquisition of full MS/MS spectra is highly inefficient. Consequently, in targeted MS assays, only the most intense and specific fragment ions are monitored, typically 3 to 5 per peptide.

The novel hybrid high-speed mass spectrometer Stellar MS follows similar design principles, but instead of a third quadrupole it uses a dual-cell linear ion trap (LIT) as a mass analyzer (Fig. 1). Conceptually, the hardware and instrument control architecture are very similar to the Thermo Tribrid Series (17) but without the Orbitrap analyzer. Simultaneous ion accumulation/fragmentation in the collision cell with mass analysis in the LIT enables acquisition rates of about 70 Hz for typical peptide scan ranges of 200 to 1400 Th and allows ion injection to take place for ∼85% of the total acquisition time. The typical mass analysis scan rate is 125 kDa/s, which gives a peak full width half maximum of about 0.7 Th at m/z 622. The resolution can be increased to about 0.5 Th at the cost of a reduced scan speed of 67 kDa/s, which would effectively half the number of targets scheduled per assay in the example shown below. The high-pressure cell of the LIT is capable of performing waveform-based synchronous precursor selection multiplexing and resonance ion activation, which are particularly useful for MS3 analysis, which can be performed at up to 40 Hz. The dual-high-energy-dynode-single-multiplier with a photo-multiplier-based final stage yields very high detector stability and longevity.

There are several advantages of parallel reaction monitoring (PRM) analysis performed on Stellar MS over the selected reaction monitoring (SRM) performed on a triple quadrupole. The first is that multiple product ions of a precursor can be accumulated and analyzed simultaneously instead of serially as in a triple quadrupole. For N product ions formed at the same collision energy, the precursor can be accumulated up to N times longer at a fixed cycle time. This can lead to an up to N-fold increase in signal, although in practice, the gain may be limited by AGC targets. In large-scale assays with very short SRM dwell times approaching the approximate 1 ms needed for hardware switching between product ions, the effective gain can even exceed N-fold due to reduced switching overhead. The second advantage is that PRM analysis enables retrospective optimization of the fragment ion transitions used for analysis, whereas SRM requires the transitions to be selected before the experiment starts. For species like peptides, which typically fragment into many product ions, PRM effectively increases the selectivity of the analysis by allowing for post-acquisition transition selection.

**Fig. 2:**
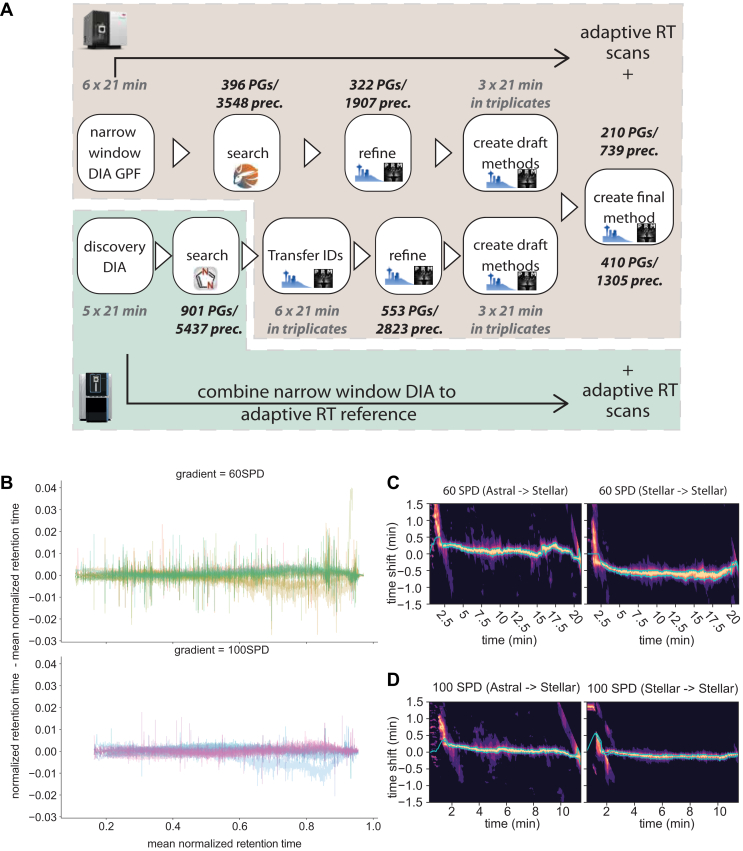
**Development of targeted assays on Stellar MS using Orbitrap Astral discovery data**. *A*, flowchart of targeted assay development for Stellar MS. Two pathways are shown: DIA on Stellar MS with gas phase fractionation (GPF) and transfer of Orbitrap Astral MS discovery data to Stellar MS. PRM Conductor software filters transitions, aiding high-quality precursor selection. *B*, line plot of the normalized retention time error against the normalized retention time for each precursor reproducibly identified in 12 consecutive technical replicates per gradient. *C and D*, contour plot of Adaptive RT cross correlations between alignment acquisition on a Stellar MS targeted run compared to either another Stellar MS run or the Orbitrap Astral MS for two different gradient lengths. Retention time shifts within ± 30 s demonstrate robust transferability and accurate real-time alignment, enabling seamless transition from discovery to targeted assays across different platforms. The band indicated in green is the estimated retention time shift used as the basis for the adjusted acquisitions in the Adaptive RT scheme.

These features open up new possibilities for targeted assay design (Fig. 2*A*). Note that the Stellar MS instrument can also acquire data in data-independent acquisition (DIA) mode, preferably by “gas phase fractionation (GPF)” to increase depth (18). From these nominal mass resolution datasets, peptides deemed to be well targetable can be selected. This has the potential to reduce optimization iterations and thereby the time to design a targeted assay, as the precursor was measurable on the setup already. Alternatively, target lists could be transferred from discovery studies performed on different instruments. Here, we were particularly interested in the transfer from the recently introduced Orbitrap Astral MS. In this example, the peptides of interest are first validated and evaluated in a set of targeted pre-screens, analyzing up to 100 precursors per minute. Based on the results, potential precursors and proteins are stepwise reduced until a single refined targeted assay is generated (see below and Experimental Procedures).

For this stepwise reduction, we utilized a new software tool called PRM Conductor that takes advantage of the Stellar MS capabilities to seamlessly build PRM assays (https://panoramaweb.org/prm_conductor.url). PRM Conductor is a Skyline external tool that uses either DIA discovery data or PRM data. Its principal functions are to filter transitions with a set of simple criteria, such as absolute and relative area, signal-to-background ratio, covariance to the median, and LC peak width. Precursors with at least a minimum number of qualifying transitions, typically three, are accepted. Qualifying precursors are placed into the PRM assays, and a user interface allows one to visualize the effect of acquisition settings. Finally, the selected precursors can be exported to an instrument method file.

Along with target filtering and selection, the PRM conductor has an integrated data compression tool. Briefly, reference spectra from periodic acquisitions, such as from DIA, are compressed and can be embedded in the instrument method file. These spectra can originate from discovery experiments on a Stellar MS, or another instrument such as an Orbitrap Exploris or Orbitrap Astral. The compressed files are used by the acquisition software in a new PRM data acquisition strategy called Adaptive RT realignment, with a principle described previously (19). The PRM method acquires extra DIA scans and periodically compares their spectra with the reference data to update the scheduled acquisition windows in real time. This can be used to reduce the scheduled acquisition window widths to around a 1 min or less without missing targets from chromatographic drift. Taken together, PRM Conductor facilitates a seamless transfer from discovery results to a PRM assay, including from one instrument to another.

Realignment at the beginning and at the end of the gradient can be particularly challenging. Typically, no peptides elute reproducibly in the first section of the gradient, which is only the case from a mean normalized retention time of 0.7 (60 SPD) or 0.15 (100 SPD). In last part of the gradient retention times again start to deviate from the mean retention time. These effects can be attributed to the peptide loading as well as the wash-out phase, with the length of each phase often dependent on the gradient design and duration (Fig. 2*B*). During the peptide loading, the wide window MS2 signal used for realignment is dominated by chemical noise while during the wash out phase, the precursors captured within the wide MS2 windows can vary due to less reliant elution times. Especially, the noise at the beginning of the gradient introduces a challenge for the adaptive RT realignment. Nonetheless, slight retention time shifts can be recovered independent of the origin of the realignment file (Fig. 2, *C* and *D*).

### Discovery DIA on Orbitrap Astral and Translation of Targets to Stellar MS

**Fig. 3:**
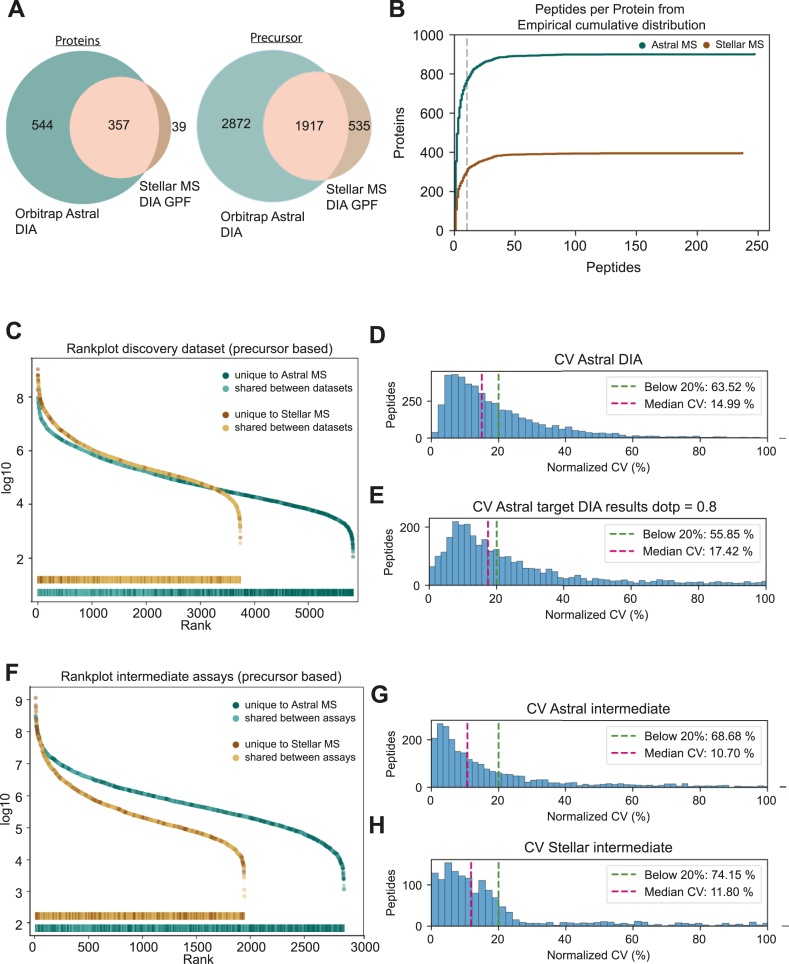
**Discovery DIA on Orbitrap Astral and Stellar MS**. *A*, precursor and protein groups identified in a single-shot Orbitrap Astral discovery DIA measurement and a Stellar MS DIA with gas phase fractionation (GPF). *B*, empirical cumulative distribution of peptides per protein identified by the Orbitrap Astral (*green*) and by the Stellar MS (*brown*). The majority of proteins is identified by 10 (*grey line*) or less peptides independent of the acquisition platform. *C*, intensity-based rank plot combined with a rugplot projection of precursors identified in the discovery datasets. Peptides identified uniquely on the Astral MS (*dark green*) or uniquely on the Stellar MS (*dark brown*) as well as shared between both platforms (*light green/light brown*). *D and E*, coefficients of variation (CVs) for close to 5000 peptides measured on Orbitrap Astral DIA acquisition and Stellar MS PRM subset measurements with a dotp above 0.8. The targeted subsets on Stellar MS show a slightly higher median CV of 17% compared to 15%. *F*, intensity based rank plot combined with a rugplot projection of precursors identified in the intermediate datasets. Peptides identified uniquely on the Astral MS (*dark green*) or uniquely on the Stellar MS (*dark brown*) as well as shared between both platforms (*light green/light brown*). *G and H*, coefficients of variation (CVs) for the intermediate assays either based on Orbitrap Astral MS or Stellar MS discovery data, acquired on Stellar MS in PRM subsets. The targeted subsets on Stellar MS show a substantially lower CV in comparison to the discovery data and a comparable CV between both assays of 10.7% for the Orbitrap Astral MS based assay and 11.8% for the Stellar MS based assay.

We compared targeted assay design based on Orbitrap Astral DIA data and Stellar MS DIA-GPF data. Both identified a broad range of peptides and protein groups, whereas the Stellar MS identifications are largely recapitulated by those identified with the Orbitrap Astral (Fig. 3*A*), with the Orbitrap Astral generating around 40% more targetable precursors and around twice as many targetable proteins. The empirical cumulative distribution of peptides per protein identified by the Orbitrap Astral and by the Stellar MS follow the same trend with the large majority of proteins measured by less than 10 peptides (Fig. 3*B*, Supplemental Fig. S1). The additionaly identified precursors are located at the lower end of the measured abundance range likely due to superior mass resolution and accuracy (Fig. 3*C*).

We split the 5437 precursor identified by the Orbitrap Astral into six subsets and remeasured them on the Stellar MS in a targeted manner. The discovery data was measured at a median CV of below 15% (63.5% below 20%, Fig. 3*D*) whereas when all precursors are targeted the median CV increases to around 20% (Supplemental Fig. S2). With a dotp (a spectral similarity-based dot product reported by Skyline) of at least 0.8 more than 60% of precursors and all the proteins remain with a slightly higher median CV of 17.4% (Fig. 3*E*). It is likely that with more subset assays, more precursors could have been validated with an overall lower CV as a substantial number of precursors are low abundant and would profit from injection times longer than accounted in this assay design (Supplemental Fig. S3). Overall, this highlights the power of the Stellar MS to cross-validate discovery DIA data from Orbitrap Astral.

Next, we evaluated both targeted assay design modalities by designing targeted assays starting from both discovery datasets in parallel. An intermediate assay, three sets of target lists each, considered all precursors measured in the Stellar GPF, as well as all precursors transferred and cross-validated from the Orbitrap Astral to the Stellar MS, which passed filtering criteria like minimum number of qualifying transitions (see Experimental Procedures). Interestingly, the precursor overlap decreases while the number of shared proteins increases. Evaluation of the covered abundance range does not reveal a trend of the shared precursors towards the higher or lower end of the abundance range. (Fig. 3*F*). Both intermediate assays achieve an overall median CV of around 11% with 70% to 75% of precursors below 20% (Fig. 3, *G* and *H*).

### Characterization of Target Lists from Orbitrap Astral and Stellar MS

**Fig. 4:**
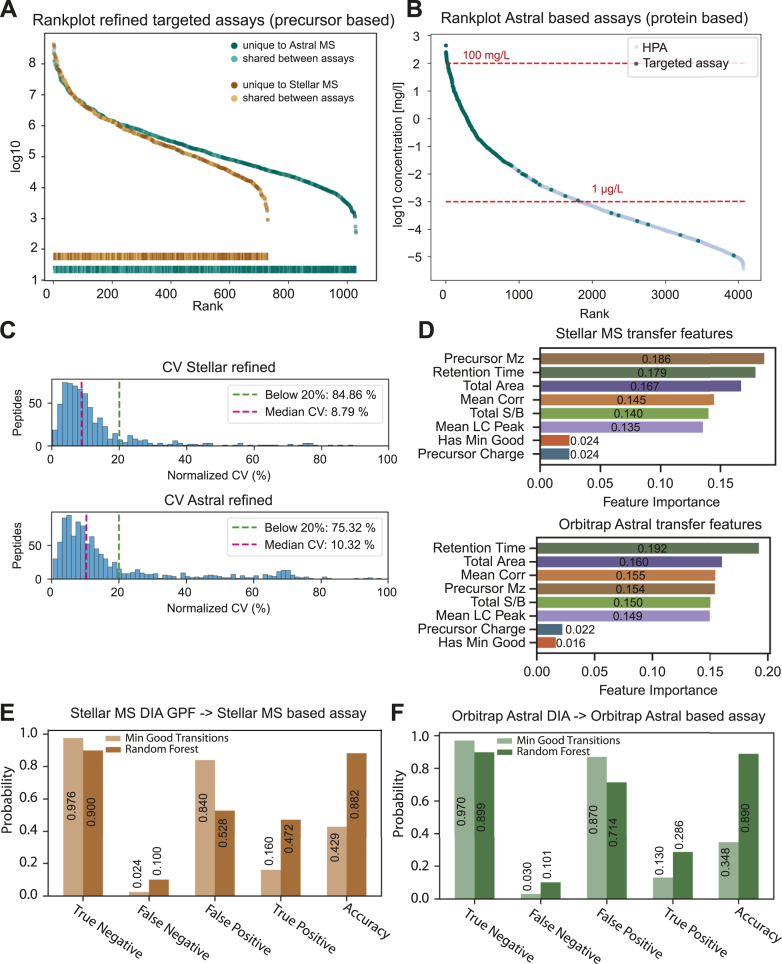
**Comparison of Orbitrap Astral discovery DIA IDs and Stellar MS GPF IDs**. *A*, intensity-based rank plot combined with a rugplot projection of precursors in the refined targeted assays. Peptides included only on the Astral MS (*dark green*) or only on the Stellar MS (*dark brown*) as well as shared between both platforms (*light green/light brown*). *B*, coverage of proteins reported in MS-based assays by the Human Protein Atlas (*light blue*) captured by the Stellar MS targeted assay based on Orbitrap Astral discovery data (*dark blue*), illustrating the broad dynamic range of protein abundances targeted. *C*, coefficients of variation (CVs) for peptides measured in Stellar MS (*top*) and Orbitrap Astral (*bottom*) based PRM assays. Despite targeting more peptides in the Orbitrap Astral-based assay, the median CVs for shared peptides remained similar. *D*, ranked feature importance for the Stellar MS-based random forest model (*top*) and Orbitrap Astral MS-based random forest model (*bottom*) used to design the PRM assay. *E and F*, performance metrics of a Stellar MS discovery-based PRM assay (*brown*) and Orbitrap Astral MS discovery-based PRM assay (*green*) designed using a minimum number of good transitions (*light brown/light green*) compared to a random forest machine learning (ML) model (*dark brown/dark green*).

In a final step, precursors are again reduced to build a single assay with the goal of targeting as many proteins as possible. The fraction of shared precursors between both assays reduces from 36% in the discovery dataset to 18% in the refined assay, while the fraction of shared proteins increased from 38% to 45%. Here, shared precursors show a trend towards the higher abundance range (Fig. 4*A*). The majority of proteins that remain in the refined assay, designed based on the Astral discovery data, are expected to have a concentration of at least 1 μg/L, according to the expected concentrations reported by the Human Protein Atlas. Targeted proteins range from 440 ug/ml (Ceruloplasmin) down to 26 ng/L (SUMO1 activating enzyme subunit 1) (Fig. 4*B*) 22 May 2024 www.proteinatlas.org/humanproteome/blood+protein/proteins+detected+in+ms). Comparing the mean total area of all precursors of a protein, as an approximation of their abundance, with the expected concentration, we found that the relationship follows a linear trend down to a concentration of 1 μg/L, after which the mean total area remains stable around the detection limit (Supplemental Fig. S4). This is likely due to an increased injection time compensating for lower precursor abundance, which in some cases increases the signal just enough to reach the detection limit.

Independent of the source datasets, both assays can be measured with a similar quantitative reproducibility, with close to 85% of precursors having a CV below 20% for the Stellar-based assay and 75% of precursors below 20% for the Astral-based assay (Fig. 4*C*).

To transition from a list of targetable peptides to a fully developed targeted assay, it is essential to select peptides that can be measured reproducibly in terms of retention time and signal intensity. As we wanted to move from selecting just a few peptides towards targeting thousands of peptides in a single assay, we needed to prioritize candidates from the discovery data in order to rapidly design our target list. We used PRM conductor for this task, a sophisticated ranking and filtering tool which selects precursors based on passing criteria like mean total area, signal to noise ratio or retention time correlation. Another commonly used criterion is to acquire a minimum number of transitions per peptide, which we use later for a simple classification criterion. Despite using the same tool with substantially overlapping information on a qualitative level - 36% at the peptide level overall, and close to 80% of all peptides discovered by the Stellar MS were also identified by the Orbitrap Astral - different precursors passed the filtering process. We investigated this disparity by training a random forest model with the purpose of predicting which peptides from our discovery data are present in the final assay. Interestingly, the precursor mass has the highest feature importance for the Stellar-based assay, while this is only the fourth most important feature in the Astral-based assay, where the retention time and total area are of higher importance (Fig. 4*D*).

Prediction accuracy for peptides included in the final assay was twice as high for both the Stellar and Astral datasets, mainly due to fewer false positives and more true positives. Especially reducing the false positive rate is an important step, as this speeds up the whole assay design procedure. (Fig. 4, *E* and *F*). Including more information and a machine learning approach thus promises further improvements of the predictability of which peptides are well targetable from all those identified in discovery experiment setups.

### ^15^N Labeled Proteins in Targeted Proteomics

Our data demonstrated that the Stellar MS is capable of targeting a large number of peptides in short gradients. To move towards absolutely quantitative assays, we next explored ^15^N-labeled protein standards from our previously established liver biomarker panels (12). Seven of these proteins (SERPINC1, QSOX1, TTR, ALDOB, ITIH4, ATRN, and SELENOP) were recombinantly produced by Absea Biotechnology at a sufficient scale for a very large number of assays (Experimental Procedures).

**Fig. 5:**
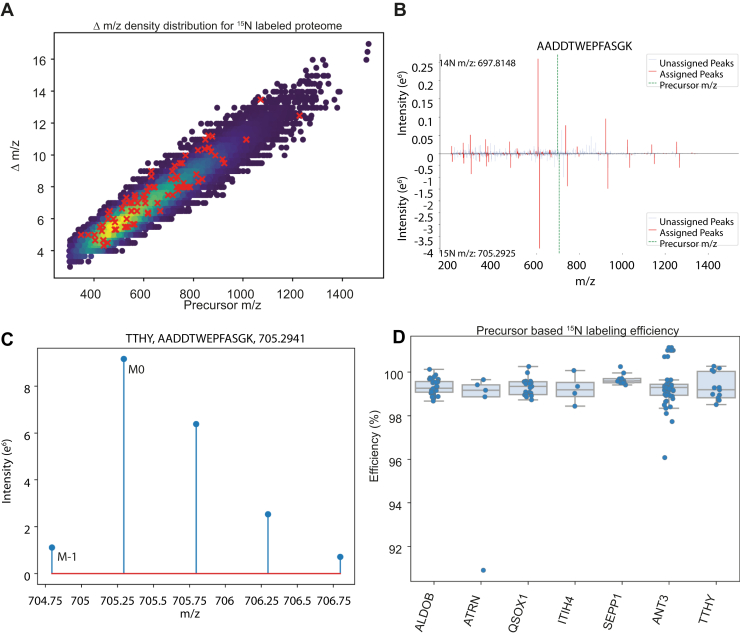
**Characterization of ^15^N-Labeled Protein Standards.***A*, distribution of mass shifts for a ^15^N-labeled total human proteome. The histogram shows the range of mass shifts calculated for tryptic peptides, reflecting the mass shift due to ^15^N across different amino acid compositions and lengths. *B*, MS2 mirror plot comparing the measured fragmentation patterns of a heavy ^15^N-labeled peptide and its light counterpart. The mirror plot illustrates the individual mass shifts between the labeled and unlabeled peptides. *C*, Extracted precursor isotope envelope of AADDTWEPFASGK. The graph shows the measured monoisotopic peak (M0) and the M-1 peak, where at least one heavy nitrogen is replaced by a light one. The degree of ^15^N incorporation for each protein is calculated by the ratio of monoisotopic (M0) and M-1 peaks, with a linear regression applied to predict labeling efficiency (Experimental Procedures). *D*, detailed view of labeling efficiency for specific proteins within the selected *panel*, confirming over 99% labeling efficiency for all analyzed proteins. This high efficiency ensures accurate quantification and reduces potential interferences in mass spectrometric analysis.

This enables the design of targeted assays based on whole ^15^N labeled protein panels where we target multiple peptides for each protein at the same time, significantly increasing the number of targets per assay compared to labeled peptide standards. Unlike C terminal stable isotope labeled synthetic peptides that have a fixed mass shift for every peptide, the mass shift of ^15^N-labeled peptides depends on their amino acid (aa) composition and length ranging from Δm/z = 4 to Δm/z = 16 in tryptic digests (Fig. 5*A*). The tryptic peptides targeted for the selected proteins cover the range of typically observed mass shifts (Fig. 5*A*, red crosses). Since every aa is labeled, both b and y fragment ions are shifted based on their nitrogen content and charge (Fig. 5*B*).

We evaluated the degree of ^15^N labeling for our target panel based on their precursor envelope. Incomplete labeling would have led to highly complex MS2 spectra with distorted fragment ion ratios depending on the position of missed labels, but this distortion was not observed. A characteristic feature of isotope contents is the ratios between the monoisotopic mass peak (M0) and its counterparts, here specifically the M-1 peak, in which one atom was not exchanged with a heavier isotope. Such an extracted isotope envelope is illustrated for the peptide AADDTWEPFASGK in Figure 5*C*. We calculated the M-1/M0 ratios for every peptide by predicting MS1 envelopes for different simulated labeling efficiencies and thereby accurately determined the labeling efficiency of every precursor using a linear regression as introduced previously (20) (Supplemental Fig. S5). Based on this procedure we estimate a degree of labeling of more than 99% for all of our proteins (Fig. 5*D*).

### Designing a Targeted Acquisition Scheme for Liver Diseases Using ^15^N-Labeled Protein Standards

With a set of high-quality protein standards in hand, we designed a targeted acquisition scheme for the measurement of the ^15^N-labeled protein panel and its light counterparts. Targeting the standards at 1 ng each in a 60 SPD gradient, we achieve a median CV of 7.9% (89% of precursors with CV below 20%) across 56 heavy precursors with an average of 14.5 data points per peak across all 112 targeted precursors (Supplemental Fig. S6). Overall, the assay achieves a median CV of 5.1% (100% with CV below 20%) across all precursors used for absolute quantification when normalized to the signal of the heavy spike-in (Supplemental Fig. S6). The limits of detection (LOD) and limits of quantification (LOQ) were as expected with the majority of LODs and LOQs being below the 0.1 ng dilution level with the lowest LOQ per protein in the low attomole range (Supplemental Fig. S7, Supplemental Table S2).

The dual linear ion trap design of the Stellar MS allows for efficient MS3 targeting of selected transitions. Applying this to the set of ^15^N labeled peptides did improve the limits of detectability in some cases (Supplemental Fig. S7), while overall the LODs and LOQs stayed similar. We speculate that this is due to better signal intensity for MS2-based targeting (Supplemental Fig. S7) and better signal-to-noise in MS3-based targeting (Supplemental Fig. S7), which plays out differently for different peptides. The targeting principle has to be selected on a peptide-to-peptide basis, and in our example, longer precursors tend to benefit more often from MS3. However, this has to be balanced carefully as MS3 targeting influences the duty cycle such that one precursor might profit at the cost of precursors eluting in the neighborhood. Here, we achieved absolute quantification of the 15N-labeled protein panel with a pure MS2-based assay. Of note, we only explored a combination of HCD followed by CID, while the instrument enables all combinations of CID and HCD.

**Fig. 6:**
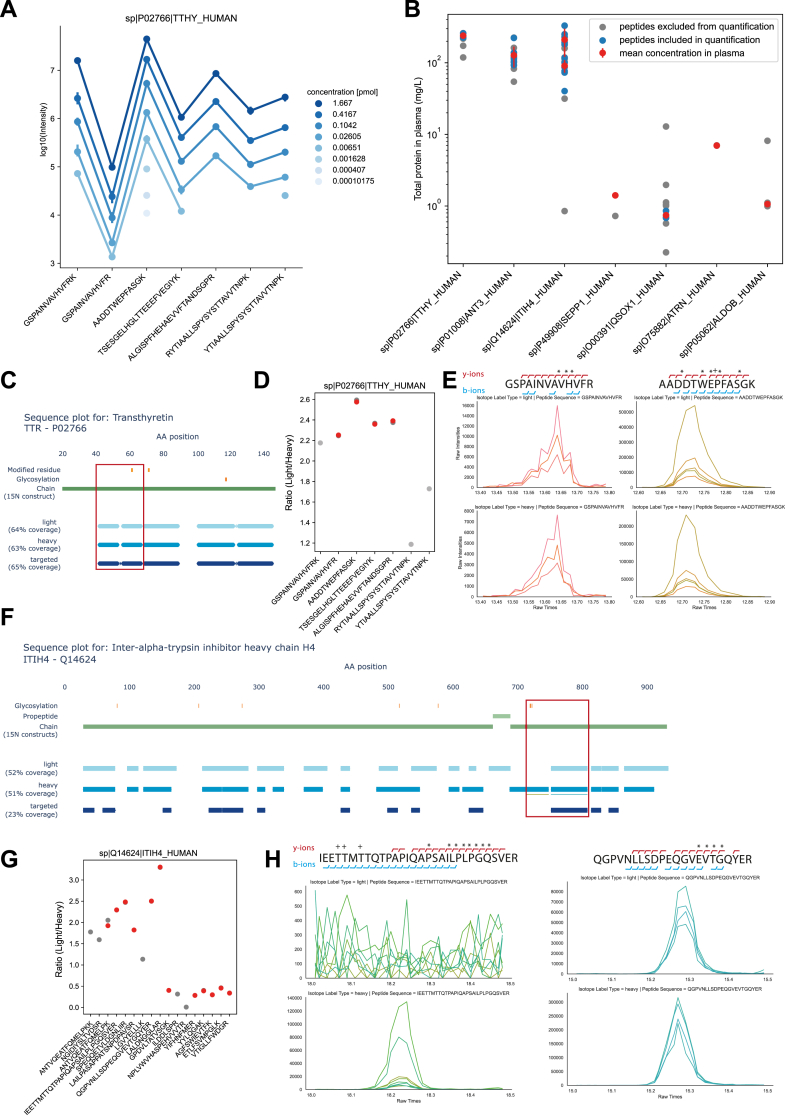
**^15^N targeting of liver panel proteins**. *A*, peptide area-based signals for peptides along transthyretin (TTHY/TTR) sequence titrated starting from 1.667 pmol (25 ng) down to 101.75 amol (1.5 pg). This graph shows the consistent detection of peptide signals corresponding to varying levels of protein spiked in. Each peptides lower limit of quantification (LOQ) is between the last two concentrations shown for the respective peptide. *B*, the mean concentration and error of each absolutely quantified protein (*red*) using a subset of peptides (*blue*) targeted in the assay. Peptides are excluded (*gray*) if they fall under the limit of quantification, have less then three interference free quantifiable fragments or if the heavy peptide signal is below a signal to noise ratio of 5. *C* and *F*, sequence plot for transthyretin and inter alpha trypsin inhibitor chain H4 (ITIH4) with annotated chains, propeptides and modifications using AlphaMap (27). The ^15^ N labeled construct corresponds to the chains of the protein. Horizontal bars for light (identified in neat plasma DIA runs, *light blue*), heavy (identified in DIA runs of ^15^N-labeled proteins spiked into neat plasma, *blue*) and targeted (peptides included in the targeted assay used for absolute quantification, *dark blue*) represent the parts of the protein sequence covered by identified peptides. Neighboring peptides are visualized without breaks. *D*, peptide area-based ratios between light and heavy versions of all TTR peptides included in the targeted assay. *Gray* peptides are excluded due to reasons described in (*B*), *dark gray* represents the intensity ratios uncorrected for the labeling efficiency (see Fig. 5), *red* represents the corrected peptide intensity ratios. *E*, extracted ion chromatograms for two neighboring peptides from TTR. *Top row*: light version of the peptide. *Bottom row*: heavy version of the peptide. Each color represents one fragment ion. ∗ marks the ions that were plotted while + marks the disease associated modification sites. *G*, peptide area-based ratios between light and heavy versions of all ITIH4 peptides included in the targeted assay. Gray peptides are excluded due to reasons described in (*B*), *dark gray* represents the intensity ratios uncorrected for the labeling efficiency (see Fig. 5), *red* represents the corrected peptide intensity ratios. *H*, Extracted ion chromatograms for three neighboring peptides from ITIH4. *Top row*: light version of the peptide. *Bottom row*: heavy version of the peptide. All matched ions are indicated above (y-ions, *red*) or below (b-ions, *blue*) the sequence and the ones highlighted with ∗ are shown in the extracted ion chromatogram.

Upon closer inspection of the peptides of each protein, their different characteristics, such as ionizabilities, were reflected in the reproducibility of the mean area measured over several replicates, leading to a characteristic protein intensity signature along the protein sequence. This signature shifts up and down as expected when varying protein amount (Fig. 6*A*, Supplemental Fig. S8).

These protein intensity signatures were largely recapitulated when we targeted the respective peptides in a plasma proteome sample where we had spiked in the labeled protein mixture in a fixed amount (1 ng/protein) (Supplemental Fig. S9). Out of the 56 heavy and light pairs targeted, we used six to monitor PTMs and missed cleavages. From the remaining 50 pairs, 32 (blue, Fig. 6*B*) were used for absolute quantification; the others were removed due to insufficient LOQ or due to low signal-to-noise ratio in the heavy channel (grey, Fig. 6*B*). We absolutely quantified the seven proteins in a range from 0.75 mg/L (QSOX1) to 240 mg/L (TTHY) according to the concentrations given by the Human Protein Atlas for MS detection in plasma (2023-04 version) (Fig. 6*B*, Table 1).

**Table 1:** Absolute quantified concentrations of the liver disease panel

Protein	LOQ [pmol]	Conc. In plasma [mg/L]	HPA conc. (By MS) [mg/L]	HPA conc. (By Immunoassay) [mg/L]	Peptides	Std	Std%
QSOX1	1.02E-05	0.74	1.8 (9×)	-	4	0.078	10.5
ATRN	0.00013	6.98	17 (8×)	-	2	0.018	0.26
ANT3	0.00012	127.3	110 (8×)	-	8	41.05	32.25
TTHY	0.00011	239.47	80 (8×)	290–400	4	13.44	5.61
ALDOB	0.00014	1.06	0.41 (9×)	-	1	-	-
SEPP1	0.00079	1.41	1.5 (9×)	0.05–4.4	1	-	-
ITIH4 (30 kDa)	0.00033	90.87	83 (8×)	0.001–0.006	5	18.2	20.02
ITIH4 (70 kDa)	8.14E-05	210.51	83 (8×)	0.001–0.006	6	89.3	42.42

Each protein quantified with a ^15^N-labeled protein standard, the lower limit of quantification (LOQ) of its most sensitively quantifiable peptide in pmol, its concentration in the plasma pool in mg/L, the concentration as found in the 2023-04 human plasma atlas build of the human protein atlas (HPA) including the uncertainty range, the plasma concentration as determined by Immunoassay if available, the number of peptides used for quantification, the absolute standard deviation and percentage error.

Targeting ^15^N-labeled protein standards instead of peptides opens possibilities beyond absolute quantification, which we illustrate with two examples. Transthyretin (TTR) is a relatively short thyroid hormone binding protein usually found as a tetramer. Our ^15^N labeled construct covers the active chain from aa 21 to 147 and 65% of the whole sequence could be targeted and measured in the heavy and light version of the protein. This included two missed cleaved forms at the front and end of the covered sequence. Additionally, three modifications are reported and two of which are validated, a carboxyglutamate (aa 62) reported in myoma (21), a glycosylation at N118 in conjunction with a V30 M mutation reported in familial amyloidotic polyneuropathy (22). The phosphorylation at S72 is only assigned by similarity (Fig. 6*C*). All three modifications fall into separate sequences covered by the targeting experiment and all are used for our absolute quantification. Of note, all heavy reference proteins were expressed in *E. coli* and as such there should be no native post-translational modifications. Of the seven peptides targeted for TTR (Fig. 6*D*) two were used to correct for missed cleavages and one was excluded falling below the LOQ (light grey). The other four ratios are corrected for ^15^N labeling efficiency on a peptide level (corrected: red, uncorrected: dark grey, see Experimental Procedures). The four corrected ratios amass to a 5.6% relative standard error, suggesting that any modifications on these peptides occur at negligible occupancy. A side-by-side comparison of unmodified GSPAINVAVHVFR and potentially modified AADDTWEPFASGK shows measurable heavy and light signals for both (Fig. 6*E*). This is to be expected for the two disease-related ones, one of which is located on AADDTWEPFASGK, as the associated diseases are unlikely to be present at a substantial proportion in the pooled plasma samples. The second example, Inter-alpha-trypsin inhibitor heavy chain H4 (ITIH4), is an acute phase protein that can be proteolytically processed by kallikrein into a heavy (70 kDa) and a light (30 kDa) chain. The heavy chain also undergoes further cleavage to remove a propeptide. Here, the heavy construct is also split in two, enabling separate quantification of two protein chains (Fig. 6, *F* and *G*).

The endogenous heavy chain is known to be glycosylated at multiple sites, with four positions having reported occupancies above 90%, and one site at amino acid 274 having below 1% occupancy (23). A peptide lacking glycosylation is detectable only at this low-occupancy site, supporting the conclusion that most glycosylated sites are not observed in their unmodified form. The light chain also contains a cluster of glycosylation sites near its N-terminus (Fig. 6*E*).

To investigate this further, we included two peptides from this region in our targeted assay. Both peptides show a strong signal in the labeled standard. While the endogenous form of QGPVNLLSDPEQGVEVTGQYER is clearly detecteable, the endogenous version of IEETTMTTQTPAPIQAPSAILPLPGQSVER is absent. This pattern strongly suggests that the absence of the endogenous signal for this peptide is not due to stochastic effects, but likely due to a glycosylation site with high occupancy that prevents detection of the unmodified form (Fig. 6*F*).

## Discussion

In this study, we developed and described a strategy for designing targeted proteomics assays using the novel hybrid high-speed mass spectrometer, Stellar MS, based on discovery DIA data from the Orbitrap Astral. We demonstrated how protein marker panels identified in discovery studies can be translated into targeted assays, enabling parallel targeting of peptides that span entire protein sequences in a native plasma digest and their ^15^N-labeled counterparts.

Our results show that a targeted assay can be designed and implemented reliably in a few steps, significantly reducing development time to just a few days. This streamlined process is advantageous not only for clinical assay development but also for the rapid validation of discovery studies. Findings from established and new studies could readily be combined and validated in a targeted manner and this could even be extended to biomarker candidates resulting from non-MS technologies. We have found that biologically relevant proteins often display very small changes in abundance between patient groups in clinical cohorts. In our case, only around 2% of the fibrosis-associated plasma proteins changed more than 1.5-fold in early fibrosis (12). Stellar MS can be used to verify those results, and with suitable standards, absolutely quantify proteins of interest. This capability provides new perspectives on plasma proteomics experiments, allowing the cross-validation of clinical results alongside research findings. Stellar MS has the potential to replace numerous single-protein biomarker assays, targeting and quantifying a wide range of protein concentrations, from hundreds of μgrams per mL to picograms per mL in a single run. This range includes many protein markers in routine clinical use. A report submitted in parallel compared the Stellar MS to a triple quadrupole MS and reported up to 10 fold better LOQs (24). Our results show that MS3-based acquisition can further reduce LOQs. While this does not apply universally to all precursors in our experiments - especially those below a peptide length of 10 amino acids — it often effectively halves the LOQ for longer ones. We conclude that a combined hybrid MS2 and MS3 assay, selecting for each precursor the acquisition scheme, would likely yield targeting assays with optimal sensitivity and specificity.

We also showed that peptide identification lists can be transferred between machine platforms. Owing to platforms like MassIVE or PRIDE, research laboratories should be able to use the search results and raw files obtained on high resolution platforms to readily develop targeted assays for their own samples. Beyond plasma proteomics, this has great potential for increasing accessibility of technically challenging fields like single cell proteomics to a broader community by using Stellar to target selected proteins or pathways based on data from a different laboratory. However, for this endeavor, the accurate prediction of high-quality targetable peptides needs improvement.

Targeting ^15^N-labeled protein panels on Stellar MS showed great promise. This labeling strategy provides whole protein signatures instead of single peptide ratios, increasing comparability between samples.

In principle, this approach enables the study of proteoforms, sequence variants, and post-translational modifications (PTMs), as the selective presence or absence of specific peptides can serve as indirect diagnostic indicators. Inconsistencies in heavy-to-light peptide ratios may reflect the presence of PTMs or mutations, even if the labeled standard does not carry such modifications.

For example, in the case of TTR, all three known modification sites appear unoccupied, as the corresponding endogenous peptides show consistent ratios relative to each other and to an unmodified reference peptide. This suggests that even in the absence of a modified heavy standard, digestion-based targeting of the full-length protein can provide indirect evidence for the presence or absence of modifications. Such information may be clinically relevant: for instance, glycosylated forms of TTR have been observed only in individuals with the V30 M mutation associated with familial amyloid polyneuropathy (FAP), a hereditary disease often requiring liver transplantation (22). In this context, monitoring for glycosylated or modified peptides could help distinguish genetic subtypes of disease. Similarly, the ITIH4 example illustrates how a single protein can be informative for multiple biological processes. Cleaved and uncleaved forms of ITIH4 have been reported in plasma under conditions such as inflammation or acute ischemic stroke (25). Our construct design allows the light and heavy chains to be quantified independently. Adding traditional peptide spike-ins targeting the propeptide region between the chains could further enable quantification of the full-length 120 kDa precursor alongside its cleaved forms.

The whole protein signature can additionally serve as a quality indicator for different sample preparation steps. Targeting more than one peptide per protein at a time also increases the range in which a protein can be accurately quantified (13). For low-abundance proteins, only peptides over a certain intensity range would be used to reduce the influence of signal-to-noise ratios at the detection limit. Some examples in which this concept could be relevant are the measurement of the C-reactive protein (CRP), which can range from a few milligrams per liter up to hundreds of milligrams in patients with inflammatory conditions, or alpha-fetoprotein (AFP), going from low nanograms up to micrograms in patients with hepatocellular carcinoma (26).

Overall, we have showcased the capabilities and potential of Stellar MS, a hybrid high-speed mass spectrometer, to advance targeted proteomics. This technology facilitates the rapid and easy transfer of knowledge from cohort-based discovery studies to clinical practice, bringing us closer to bridging the gap between research and patient care.

## Data Availability

The raw mass spectrometry data have been deposited in the public proteomics repository MassIVE (MSV000095051) and on Panorama (https://panoramaweb.org/StellarMS_15N_labeledproteins.url) for reviewer access. This data will be made public upon acceptance of the manuscript.

## Supplemental Data

This article contains supplemental data.

## Conflict of Interest

The authors declare the following financial interests/personal relationships, which may be considered as potential competing interests: PR, SH, and CJ are employees of Thermo Fisher Scientific. PL is an employee of Absea Biotechnology. MM is an indirect investor in Evosep.

## Supplemental Data

Supplements

Dataset description

Supplemetal Table 2
