# Supplementary material for: A Novel Hybrid High-Speed Mass Spectrometer Allows Rapid Translation From Biomarker Candidates to Targeted Clinical Tests Using 15N-Labeled Proteins
Source: Mol Cell Proteomics. 2025 Aug 13;24(9):101050. doi: 10.1016/j.mcpro.2025.101050 (PMC12455101; doi:10.1016/j.mcpro.2025.101050)
Supplement: Supplements [file mmc1.pdf]

Supplementary Tables

Supplementary Table 1: ***15N labeled protein panel.***

Gene	Uniprot ID
SERPINC1	P01008
QSOX1	O00391
TTR	P02766
ALDOB	P05062
ITIH4	Q14624
ATRN	O75882
SELENOP	P49908

Supplementary Figures

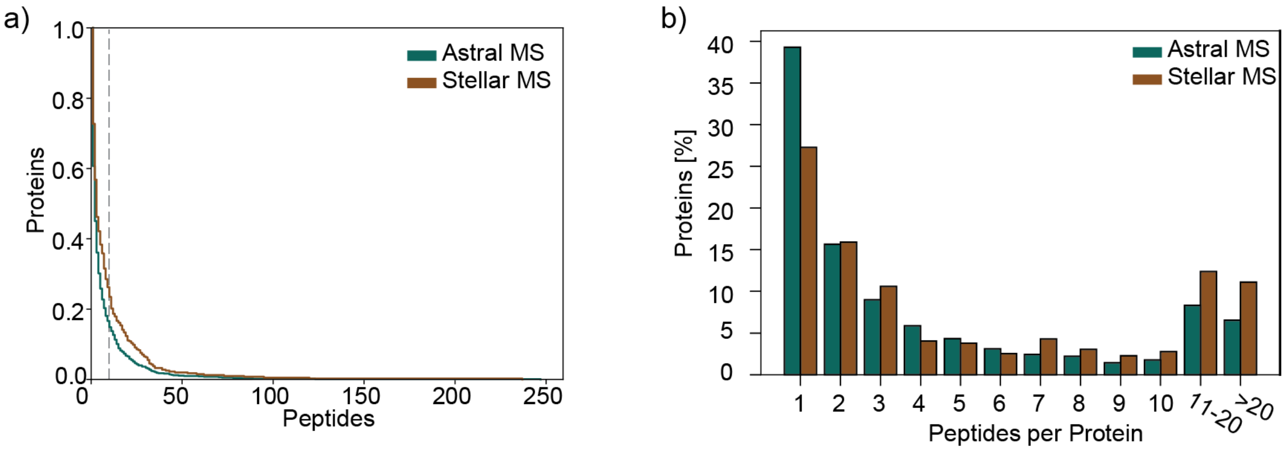

Supplementary Figure 1: ***Distribution of peptides identified per Protein from discovery experiments.***

A) *Empirical cumulative distribution of peptides per protein for discovery datasets acquired with the Orbitrap Astral MS (green) and Stellar MS (brown). The light grey dashed line marks 10 peptides per protein.*

B) *Bar chart representation of how frequently proteins are represented by one to ten peptides, between 10 and 20 and by more than 20 peptides. Close to 80% of the proteins are discovered with 10 or less peptides on the Orbitrap Astral MS (brown) and close to 70% with 10 or less peptides on the Stellar MS (green).*

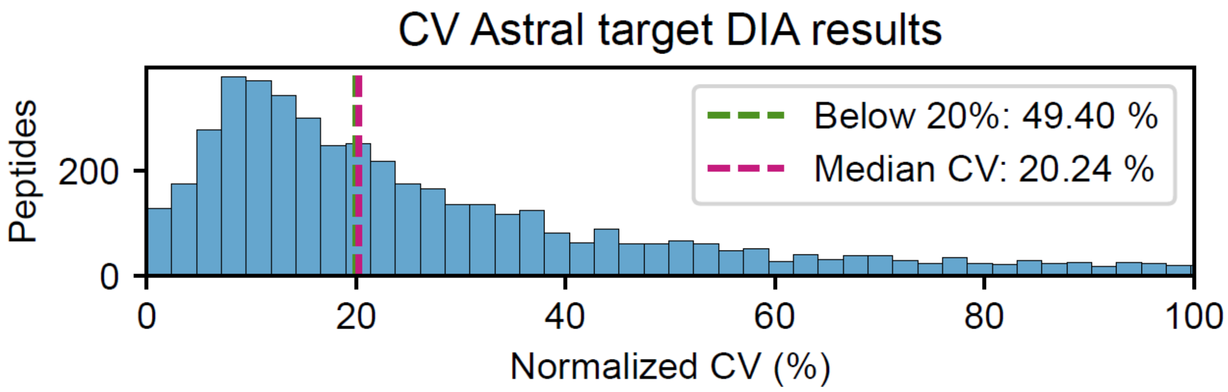

Supplementary Figure 2: ***Coefficient of variation for close to 5000 peptides targeted in neat plasma.***

Coefficient of variation for all peptides discovered in neat plasma using the Orbitrap Astral MS and subsequently targeted on the Stellar MS in PRM subsets.

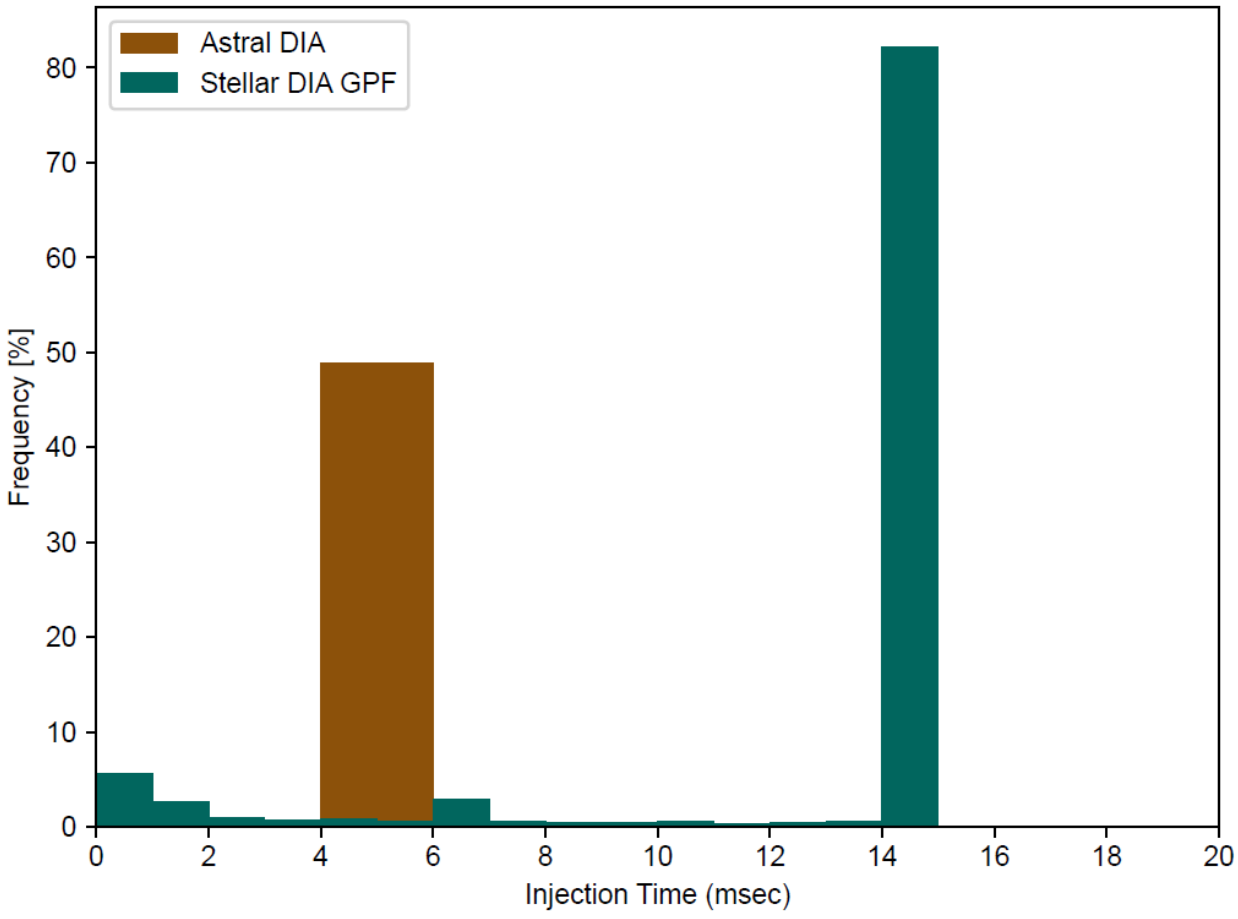

Supplementary Figure 3: ***Scan wise injection times form the discovery DIA experiments.***

Comparison of scan wise injection times for five Orbitrap Astral MS DIA single shot acquisitions (brown) and six DIA gas phase fractionation acquisitions measured on the Stellar MS (green). Most of the time, the injection time is close to the maximum injection time set for the respective experiments.

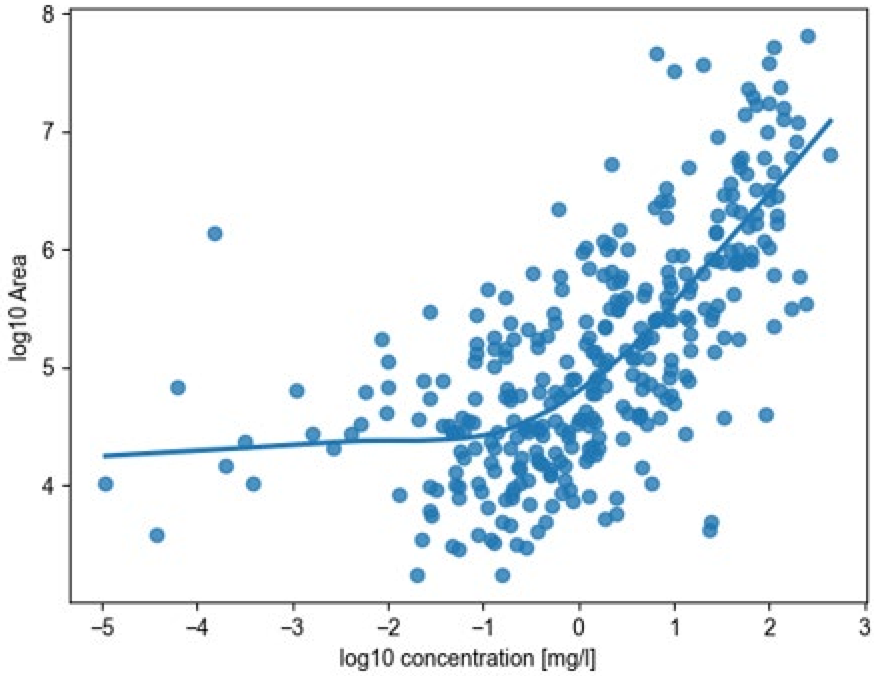

Supplementary Figure 4: ***Total Area measured per protein against their assigned concentration as translated from the human protein atlas.***

The total area measured for each protein in the refined targeted assay is plotted against the protein concentration as assigned by estimates obtained from the human protein atlases blood plasma dataset (04-2023). Up until the detection limit, a linear trend between concentration and Area is observable.

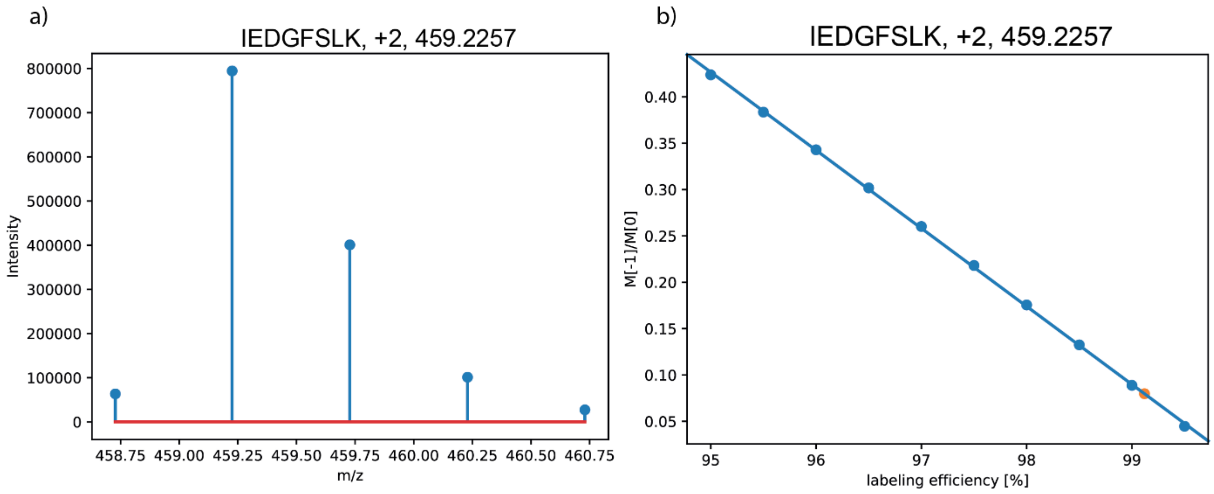

Supplementary Figure 5: ***15N labeling efficiency estimation using extracted precursor envelopes.***

A) Extracted precursor envelope from a DDA acquisition of 15N labeled IEDGFSLK, +2.

B) M-1/M0 ratios for different simulated labeling efficiencies (blue) and the extracted M-1/M0 ratio for IEDGFSLK, +2 (orange). The labeling efficiency of IEDGFSLK is estimated using a linear regression. This is repeated for all extracted precursor envelopes to estimate an overall labeling efficiency.

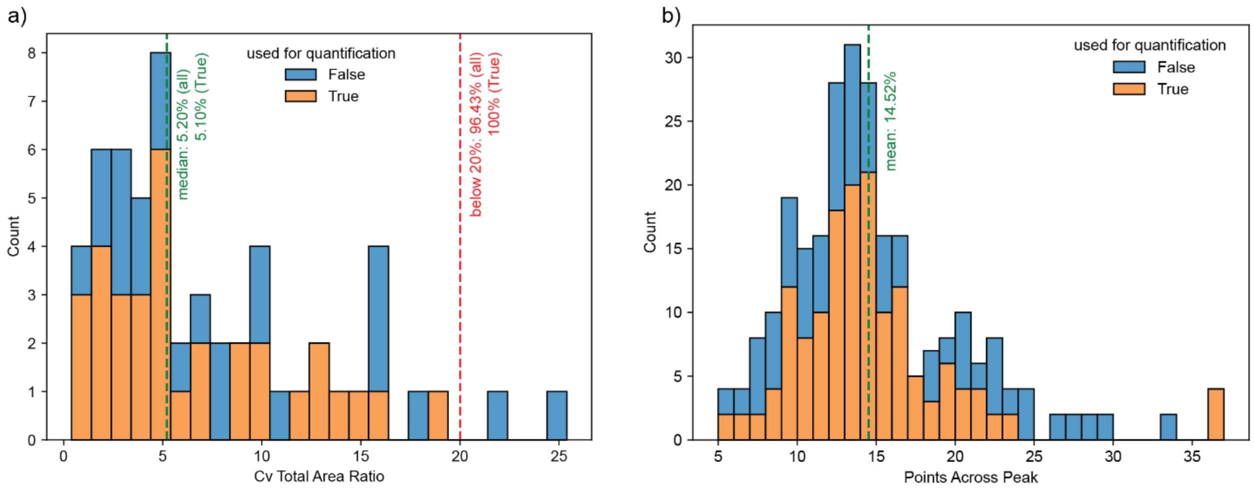

Supplementary Figure 6: ***Coefficient of Variation and data points per peak for 15N labeled protein targeting assays.***

A) Stacked histogram of total area ratio coefficient of variations (Cv) for precursors targeted in the 15N labeled protein targeted assay. All together have a median CV of 5.2% with 96% having a CV below 20%. All peptides used for quantification (orange) have a CV below 20%. All peptides used for quantification are shown in orange, the ones only used for identification and validation in blue.

B) Stacked histogram of measured datapoints per peak for 60 SPD MS2 based targeted assay of 15N labeled protein biomarker panel. All peptides used for quantification are shown in orange, the ones only used for identification and validation in blue.

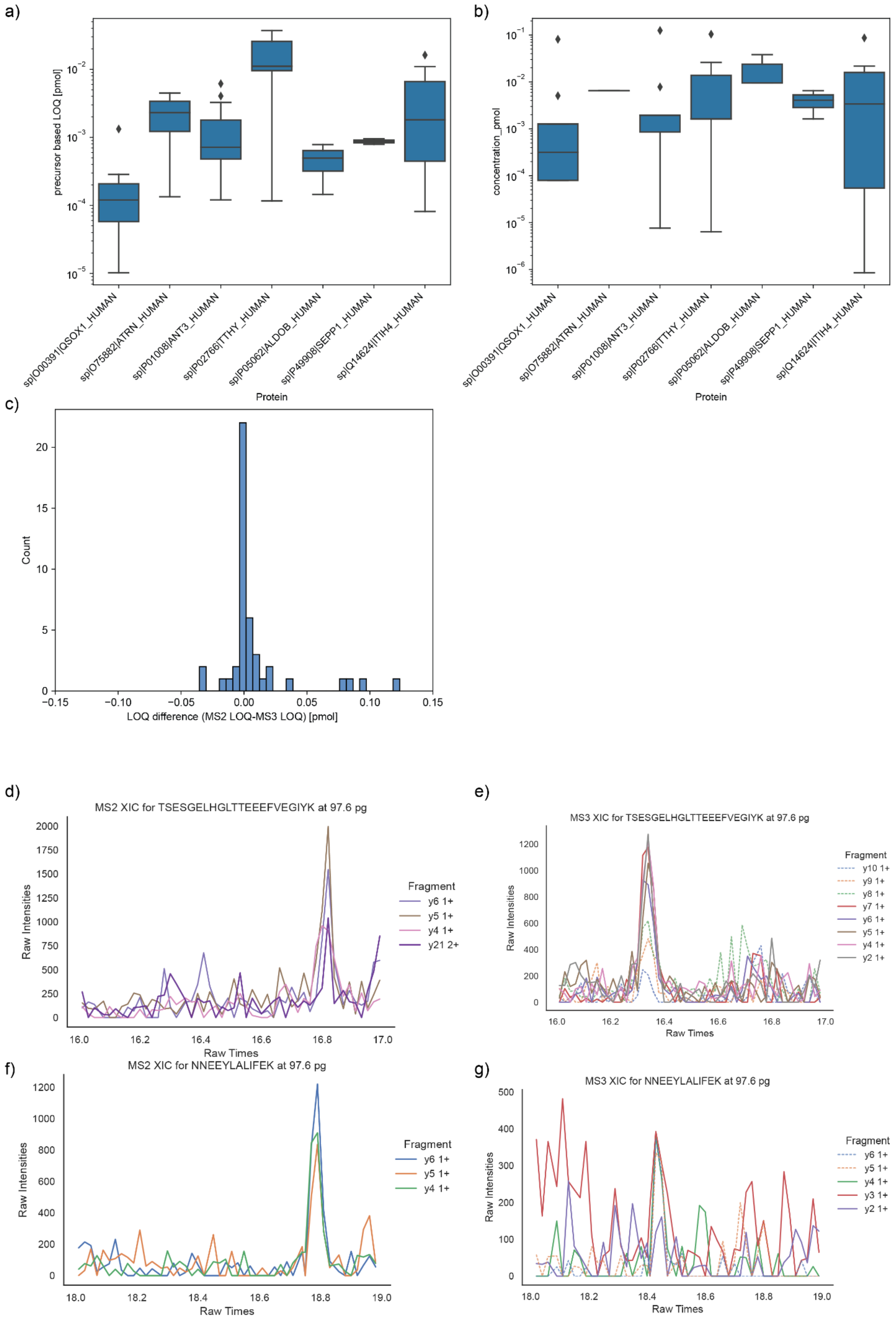
a)
precursor based LOQ [pmol]
Protein
sp|O00391|QSOX1_HUMAN
sp|O75882|ATRN_HUMAN
sp|P01008|ANT3_HUMAN
sp|P02766|TTHY_HUMAN
sp|P05062|ALDOB_HUMAN
sp|P49908|SEPP1_HUMAN
sp|Q14624|ITIH4_HUMAN
b)
concentration_pmol
Protein
c)
Count
LOQ difference (MS2 LOQ-MS3 LOQ) [pmol]
d)
MS2 XIC for TSESGELHGLTTEEEFVEGIYK at 97.6 pg
Raw Intensities
Raw Times
Fragment
y6 1+
y5 1+
y4 1+
y21 2+
e)
MS3 XIC for TSESGELHGLTTEEEFVEGIYK at 97.6 pg
Raw Intensities
Raw Times
Fragment
y10 1+
y9 1+
y8 1+
y7 1+
y6 1+
y5 1+
y4 1+
y2 1+
f)
MS2 XIC for NNEEYLALIFEK at 97.6 pg
Raw Intensities
Raw Times
Fragment
y6 1+
y5 1+
y4 1+
g)
MS3 XIC for NNEEYLALIFEK at 97.6 pg
Raw Intensities
Raw Times
Fragment
y6 1+
y5 1+
y4 1+
y3 1+
y2 1+

Supplementary Figure 7: ***LOQs of 15N labeled targeting assays.***

A) Boxplot of the limits of quantification experimentally determined for all precursors targeted in the 15N assay per protein using a MS2 targeting scheme.

B) Boxplot of the limits of quantification experimentally determined for all precursors targeted in the 15N assay per protein using a MS3 targeting scheme.

C) Difference between the MS3 and MS2 based LOQs per precursor (left lower in MS3 assay, right of 0 lower in MS2 assay)

D-E) Extracted ion chromatogram of TSEGELHGLTTEEEFVEGIYK measured at 97.6 pg in MS2 and MS3 targeting mode. This is an example for a peptide with lower LOQ in MS3 mode, and the transitions used for secondary fragmentation are highlighted in dashed lines.

F-G) Extracted ion chromatogram of NNEEYLALIFEK measured at 97.6 pg in MS2 and MS3 targeting mode. This is an example for a peptide with lower LOQ in MS2 mode, and the transitions used for secondary fragmentation are highlighted in dashed lines.

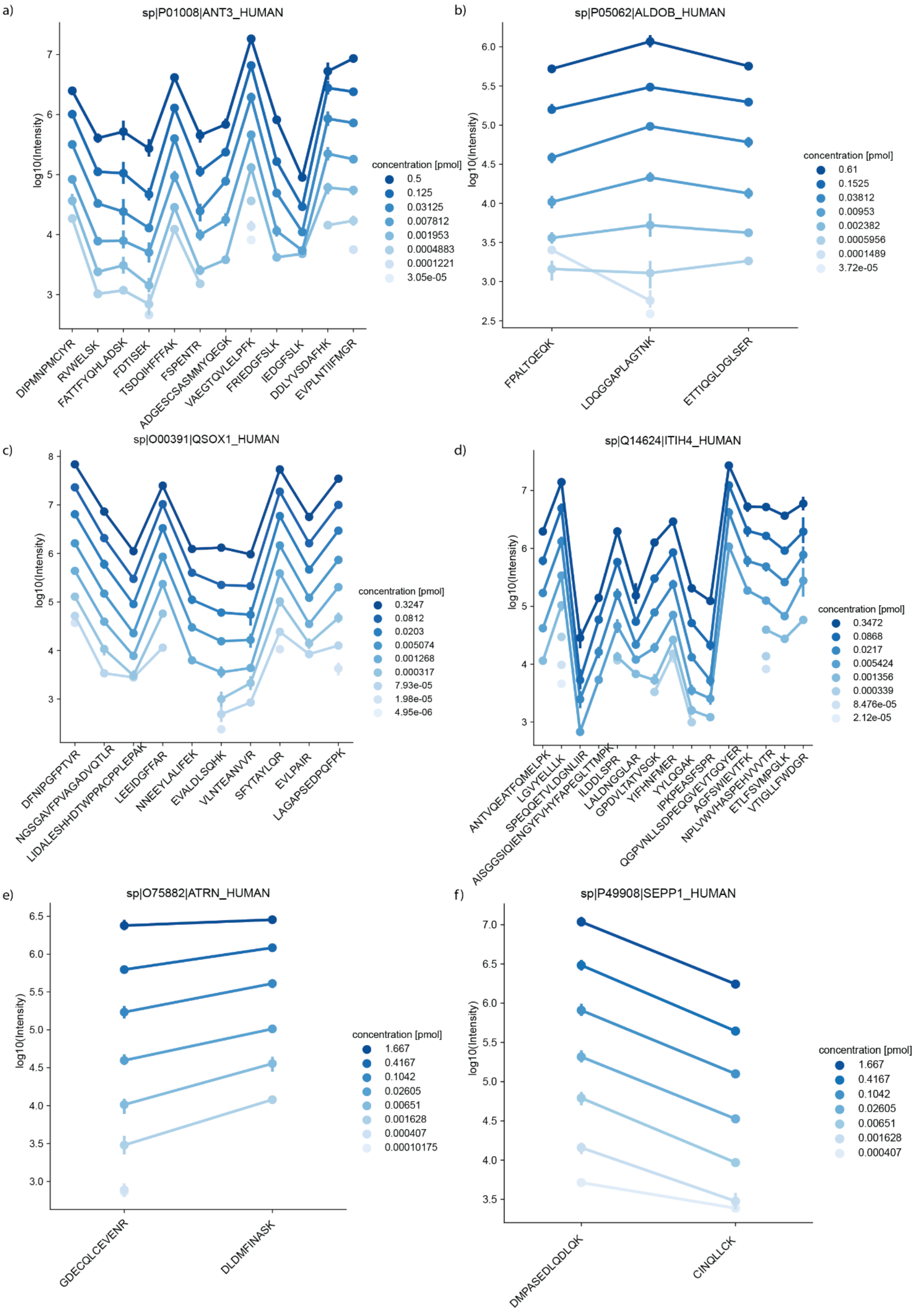
a)
sp|P01008|ANT3_HUMAN
log10(Intensity)
concentration [pmol]
0.5
0.125
0.03125
0.007812
0.001953
0.0004883
0.0001221
3.05e-05
DIPMNPMCIYR
RVWELSK
FATTFYQHLADSK
FDTISEK
TSDQIHFFFAK
FSPENTR
ADGESCSASMMYQEGK
VAEGTQVLELPFK
FRIEDGFSLK
IEDGFSLK
DDLYVSDAFHK
EVPLNTIIFMGR
b)
sp|P05062|ALDOB_HUMAN
log10(Intensity)
concentration [pmol]
0.61
0.1525
0.03812
0.00953
0.002382
0.0005956
0.0001489
3.72e-05
FPALTQEQK
LDQGGAPLAGTNK
ETTIQGLDGLSER
c)
sp|O00391|QSOX1_HUMAN
log10(Intensity)
concentration [pmol]
0.3247
0.0812
0.0203
0.005074
0.001268
0.000317
7.93e-05
1.98e-05
4.95e-06
DFNIPGFPTVR
NGSGAVFPVAGADVQTLR
LIDALESHHDTWPPACPPLEPAK
LEEIDGFFAR
NNEEYLALIFEK
EVALDLSQHK
VLNTEANVVR
SFYTAYLQR
EVLPAIR
LAGAPSEDPQFPK
d)
sp|Q14624|ITIH4_HUMAN
log10(Intensity)
concentration [pmol]
0.3472
0.0868
0.0217
0.005424
0.001356
0.000339
8.476e-05
2.12e-05
ANTVQEATFQMELPK
LGVYELLLK
SPEQQETVLDGNLIIR
AISGGSIQIENGYFVHYFAPEGLTTMPK
ILDDLSPR
LALDNGGLAR
GPDVLTATVSGK
YIFHNFMER
YYLQGAK
IPKPEASFSPR
QGPVNLLSDPEQGVEVTGQYER
AGFSWIEVTFK
NPLVWVHASPEHVVVTR
ETLFSVMPGLK
VTIGLLFWDGR
e)
sp|O75882|ATRN_HUMAN
log10(Intensity)
concentration [pmol]
1.667
0.4167
0.1042
0.02605
0.00651
0.001628
0.000407
0.00010175
GDECQLCEVENR
DLDMFINASK
f)
sp|P49908|SEPP1_HUMAN
log10(Intensity)
concentration [pmol]
1.667
0.4167
0.1042
0.02605
0.00651
0.001628
0.000407
DMPASEDLQDLQK
CINQLLCK

Supplementary Figure 8: ***Titration profiles of 15N labeled proteins.***

A-F Peptide area-based signals for peptides along the sequences titrated starting from 25 ng. This graph shows the consistent detection of peptide signals corresponding to varying levels of protein spiked in. Each peptides lower limit of quantification (LOQ) is between the last two concentrations shown for the respective peptide.

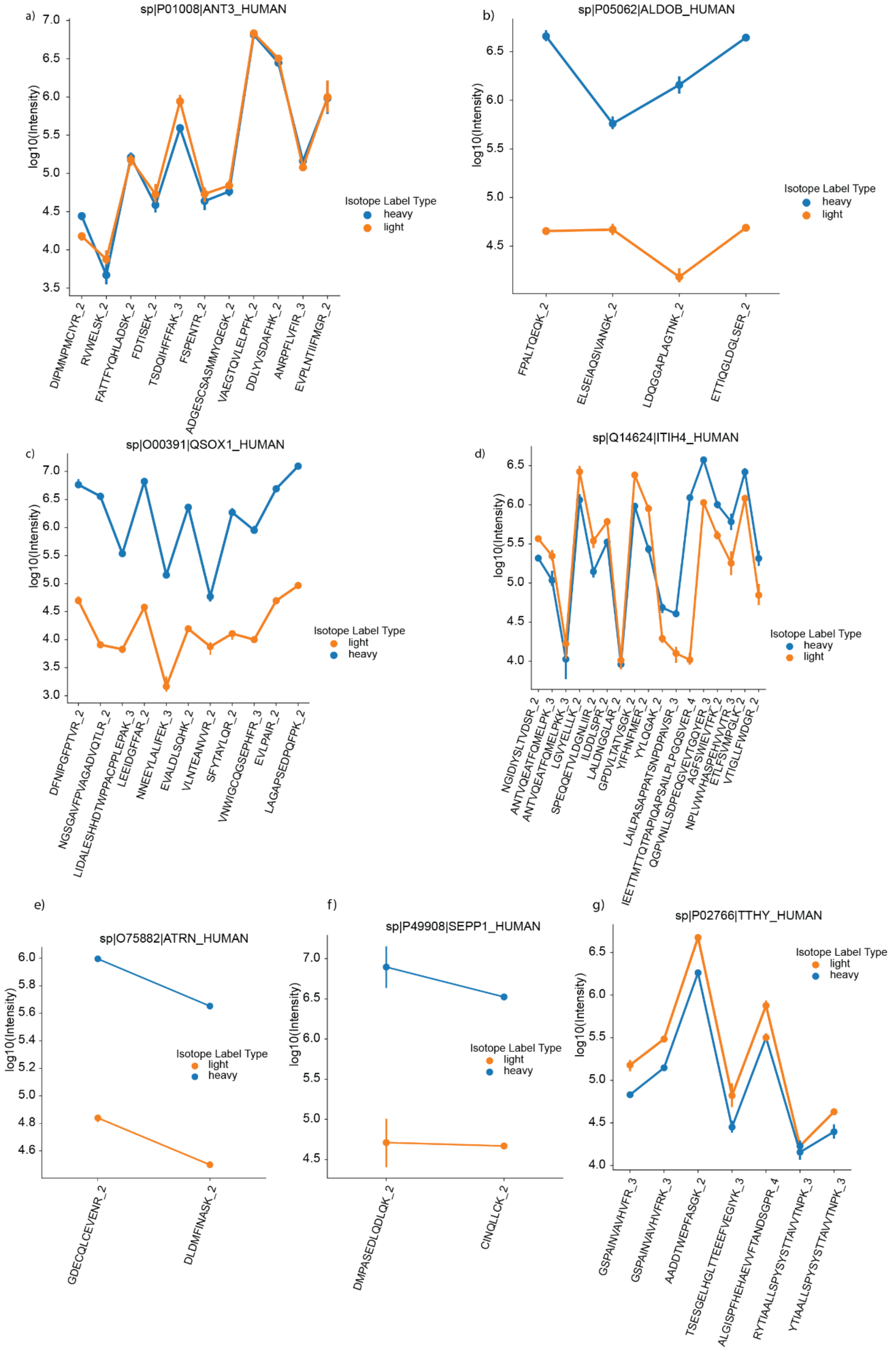

a)
sp|P01008|ANT3_HUMAN
log10(Intensity)
Isotope Label Type
heavy
light
DIPMNPMCIYR_2
RVWELSK_2
FATTFYQHLADSK_2
FDTISEK_2
TSDQIHFFFAK_3
FSPENTR_2
ADGESCSASMMYQEGK_2
VAEGTQVLELPFK_2
DDLYVSDAFHK_2
ANRPFLVFIR_3
EVPLNTIIFMGR_2
b)
sp|P05062|ALDOB_HUMAN
log10(Intensity)
Isotope Label Type
heavy
light
FPALTQEQK_2
ELSEIAQSIVANGK_2
LDQGGAPLAGTNK_2
ETTIQGLDGLSER_2
c)
sp|O00391|QSOX1_HUMAN
log10(Intensity)
Isotope Label Type
light
heavy
DFNIPGFPTVR_2
NGSGAVFPVAGADVQTLR_2
LIDALESHHDTWPPACPPLEPAK_3
LEEIDGFFAR_2
NNEEYLALIFEK_3
EVALDLSQHK_2
VLNTEANVVR_2
SFYTAYLQR_2
VNWIGCQGSEPHFR_3
EVLPAIR_2
LAGAPSEDPQFPK_2
d)
sp|Q14624|ITIH4_HUMAN
log10(Intensity)
Isotope Label Type
heavy
light
e)
sp|O75882|ATRN_HUMAN
log10(Intensity)
Isotope Label Type
light
heavy
GDECQLCEVENR_2
DLDMFINASK_2
f)
sp|P49908|SEPP1_HUMAN
log10(Intensity)
Isotope Label Type
light
heavy
DMPASEDLQDLQK_2
CINQLLCK_2
g)
sp|P02766|TTHY_HUMAN
log10(Intensity)
Isotope Label Type
light
heavy
GSPAINVAVHVFR_3
GSPAINVAVHVFRK_3
AADDTWEPFASGK_2
TSESGELHGLTTEEEFVEGIYK_3
ALGISPFHEHAEVVFTANDSGPR_4
RYTIAALLSPYSYSTTAVVTNPK_3
YTIAALLSPYSYSTTAVVTNPK_3

Supplementary Figure 9: ***Peptide intensity traces for light and heavy precursors targeted with a fixed spike in of 1ng ^{15}N labeled standard per protein***

A-G Precursor intensities measured in the targeted assay plotted in order of the primary aa sequence of each protein targeted. The heavy version of the protein (blue) and the light version of the protein (orange) generate largely parallel intensity traces.
